# The N-terminal Region of the Atypical Chemokine Receptor ACKR2 Is a Key Determinant of Ligand Binding*

**DOI:** 10.1074/jbc.M113.534545

**Published:** 2014-03-18

**Authors:** Kay D. Hewit, Alasdair Fraser, Robert J. B. Nibbs, Gerard J. Graham

**Affiliations:** From the Chemokine Research Group, Institute of Infection, Immunity and Inflammation, University of Glasgow, Glasgow G12 8TA, United Kingdom

**Keywords:** 7-Helix Receptor, Chemokines, Flow Cytometry, Inflammation, Mutagenesis

## Abstract

The atypical chemokine receptor, ACKR2 is a pivotal regulator of chemokine-driven inflammatory responses and works by binding, internalizing, and degrading inflammatory CC-chemokines. ACKR2 displays promiscuity of ligand binding and is capable of interacting with up to 14 different inflammatory CC-chemokines. Despite its prominent biological role, little is known about the structure/function relationship within ACKR2, which regulates ligand binding. Here we demonstrate that a conserved tyrosine motif at the N terminus of ACKR2 is essential for ligand binding, internalization, and scavenging. In addition we demonstrate that sulfation of this motif contributes to ligand internalization. Furthermore, a peptide derived from this region is capable of binding inflammatory chemokines and inhibits their interaction with their cognate signaling receptors. Importantly, the peptide is only active in the sulfated form, further confirming the importance of the sulfated tyrosines for function. Finally, we demonstrate that the bacterial protease, staphopain A, can cleave the N terminus of ACKR2 and suppress its ligand internalization activity. Overall, these results shed new light on the nature of the structural motifs in ACKR2 that are responsible for ligand binding. The study also highlights ACKR2-derived N-terminal peptides as being of potential therapeutic significance.

## Teaser

**Background:** ACKR2 is an atypical chemokine receptor that is biologically distinct from conventional chemokine receptors.

**Results:** An N-terminal sulfated tyrosine motif is essential for ligand binding by ACKR2.

**Conclusion:** The structure/function basis for ligand binding by ACKR2 is similar to that of conventional chemokine receptors.

**Significance:** ACKR2 N-terminal peptides may be therapeutically useful as pan-chemokine blockers in inflammatory pathologies.

## Introduction

Chemokines are the principal regulators of *in vivo* leukocyte migration and are defined by the presence of variations on a conserved cysteine motif in their mature sequences (1), and the large chemokine family is divided into four subfamilies (CC, CXC, XC and CX3C) according to the specific nature of this motif. Mammals have ∼45 chemokines and 18 receptors that involve themselves, in sometimes very complex ways, in regulating *in vivo* leukocyte migration. Given this complexity, it is common to simplify chemokine, and chemokine receptor, biology by referring to them as being either homeostatic or inflammatory according to the *in vivo* contexts in which they predominantly function (2, 3). Thus chemokines involved in basal trafficking of leukocytes into and out of peripheral tissues and secondary lymphoid organs are referred to as homeostatic. They are typically expressed at discrete tissue locales and by discrete cell types. In contrast, inflammatory chemokines and their receptors are largely involved in responding to tissue insults, injuries, or infections. Inflammatory chemokines are not expressed at high levels at steady state but are rapidly and substantially transcriptionally activated following an inflammatory insult. These chemokines then attract inflammatory leukocytes bearing their cognate receptors, and these cells remove pathogens, engulf debris, and assist in the process of tissue repair.

In addition to the 18 signaling receptors for chemokines (4), there also exists a small subfamily of atypical chemokine receptors (ACKRs)^2^ that are characterized by an inability to mount classical receptor signaling following ligand binding (4–8). This subfamily currently comprises four receptors namely the Duffy antigen receptor for chemokines (DARC/ACKR1), D6/ACKR2, CXCR7/ACKR3, and CCRL1/ACKR4. We have been particularly interested in ACKR2, which was previously known as D6 (5). This receptor binds essentially all inflammatory CC-chemokines with high affinity but does not mount classical signaling responses following ligand binding (9–11). We and others have demonstrated that ACKR2 is a highly efficient binder, internalizer, and scavenger of inflammatory CC-chemokines (12, 13). In essence, therefore, ACKR2 plays a role in removing chemokines from inflamed sites. ACKR2 is expressed in barrier tissues including the skin, gut, and lung, as well as in the syncytiotrophoblast layer of the placenta (14–16). In adult tissues ACKR2 is prominently expressed on lymphatic endothelial cells (16, 17), although expression has also been reported on leukocytes (17–21) and keratinocytes (22). In keeping with its chemokine scavenging role, numerous *in vivo* studies utilizing ACKR2-deficient mice have demonstrated the fundamental importance of ACKR2 for the resolution of inflammatory responses (15, 23–28). Although it was initially assumed that this involved the scavenging and degradation of chemokines throughout an inflamed area, it now appears that ACKR2 plays a more subtle role in this context by minimizing inflammatory leukocyte interaction with lymphatic endothelial cell surfaces and therefore ensuring the “openness” of lymphatic channels (29–31). Thus in ACKR2-deficient mice, lymphatic vessels become congested by inappropriate association with inflammatory leukocytes, and this impairs drainage of fluid, cytokines, chemokines, and cells from inflamed sites thus accounting for the impaired resolution of the inflammatory response.

Notably, despite having been clearly demonstrated to be important in a range of *in vivo* contexts, little is known about the structure/function relationships within ACKR2 that contribute to chemokine binding. With the signaling chemokine receptors, a number of regions are known to be involved in ligand binding. Prominent among these is the N terminus and, in particular, a sulfated tyrosine motif in this region (32–41, 43). The purpose of the present study was to determine whether mechanisms important for chemokine interactions with conventional chemokine receptors are conserved in the related atypical receptor ACKR2.

In this study, we demonstrate the essential importance of sulfated tyrosine residues at the N terminus of ACKR2 for chemokine binding and internalization. In addition, we provide evidence that a peptide generated from the N terminus of ACKR2 is capable of neutralizing the *in vitro* activities of ACKR2 ligands. These data therefore highlight the N terminus as a key regulator of ligand binding by ACKR2 and suggest that peptides derived from this region may have anti-inflammatory therapeutic potential.

## EXPERIMENTAL PROCEDURES

### 

#### 

##### Antibodies

Antibodies used in this study, along with details of suppliers, are listed in Table 1.

**TABLE 1:** **Antibodies used in this study**

Antibody	Supplier	Use
Anti-HA tag antibody	Abcam	Flow cytometry
Anti Hu ACKR2 (clone 4A5)	In house	Western blotting
Anti-CCBP2 (ACKR2, human)	Sigma	Western blotting for detection of N-terminal truncated ACKR2
Anti-sulfotyrosine	Millipore	Western blotting
Anti-TPST1 (human)	Abcam	Western blotting
Anti-TPST2 (human)	Abcam	Western blotting
Anti-β-tubulin	Cell Signalling	Western blotting
Streptavidin-PE	R&D Systems	Western blotting
FITC-labeled anti-mouse	R&D Systems	Flow cytometry
Biotinylated mouse IgG1-isotype control	R&D Systems	Flow cytometry
Mouse IgG2a isotype control	Dako	Flow cytometry
Mouse IgG3 isotype control	Amersham	Flow cytometry
Anti-beta-actin	Cell Signalling	Western blotting

##### Cell Culture, Transfection, and PCR

HEK 293 cells were maintained in DMEM (Sigma-Aldrich) plus 10% FCS, 4 mm glutamine, and streptomycin and penicillin (all from Invitrogen). The human acute monocytic leukemia cell line, THP-1, was maintained in RPMI 1640 (Sigma), 10% FCS, 4 mm glutamine, and streptomycin and penicillin (all from Invitrogen). All cultures were incubated at 37 °C with 5% CO_2_ and 95% humidity. Human dermal-derived lymphatic endothelial cells (17), human keratinocytes (22), BeWo (14) cells, CHO cells (17), and monocytes (19) were cultured as described previously. Note that transfected cell lines were used for the majority of the studies reported in this paper. The reasons for this were to ensure consistency with the ACKR2 mutant data (which by necessity had to be done in transfected cells) and because the primary cells responsible for physiological expression of ACKR2 *in vivo* are problematic. Specifically, lymphatic endothelial cells lose ACKR2 expression *in vitro* (17), and the placental BeWo cell line displays a lengthy doubling time (14), making the studies we report impractical in this cell background.

##### Generation of Epitope-tagged Human ACKR2 (HA-ACKR2) and Related Mutants

Nucleotides encoding an N-terminal HA tag (MYPYDVPDYAG) were introduced into human ACKR2 cDNA by PCR to generate HA-ACKR2 as described (44). Products were verified by sequencing (MWG Operon, London, UK) and cloned into pcDNA3.1 (MWG Operon). The HA-ACKR2 pcDNA3.1 plasmid was mutated using the QuikChange lightning multisite-directed mutagenesis kit (Stratagene and Agilent Technologies), and primers encoding tyrosine to phenylalanine mutations were designed and used to generate point mutations in the ACKR2 sequence (primers detailed in Table 2). All primers were from IDT (Interleuvenlaan, Belgium) and were designed with the primer design guidelines detailed in the kit instruction manual.

**TABLE 2:**
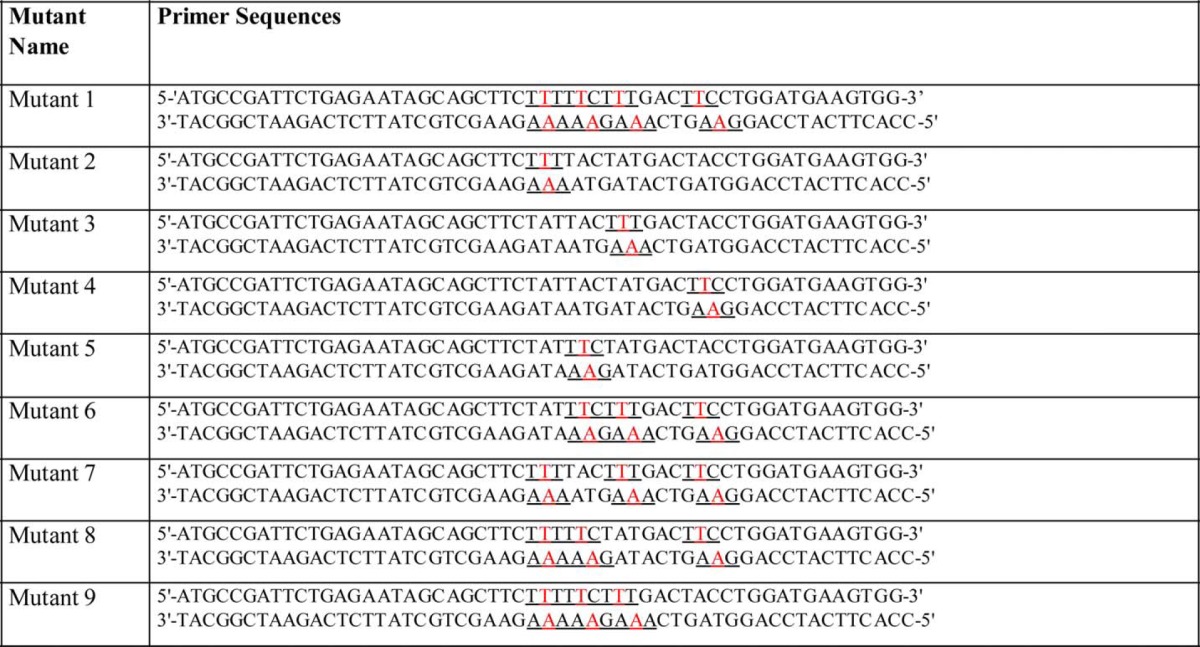
**Primers used for the generation of the ACKR2 mutants**

##### Generation of ACKR2 Transfected HEK 293 Cells

Plasmids were stably transfected into HEK 293 cells using the Effectene® transfection reagent kit (Qiagen). Stable transfectants were selected in 1.6 mg/ml G418 (Promega, Southampton, UK). High ACKR2-expressing cells were separated from low expressing and nonexpressing cells by adding anti-HA biotin (Miltenyi Biotec) to cells and mixing with anti-biotin MicroBeads (Miltenyi Biotec). Cells were run through MACS separation columns (Miltenyi Biotec) attached to a MidiMACS separator (Miltenyi Biotec), and eluates were collected. Expression of ACKR2 was verified by flow cytometry using anti-ACKR2 monoclonal antibody and FITC-labeled secondary antibody (R&D Systems) or alternatively biotinylated anti-HA antibody (Miltenyi Biotec) and phycoerythrin-streptavidin-labeled secondary antibody (R&D Systems).

##### Chemokine Uptake Assays

Either CCL2 or CCL22 (labeled with Alexa Fluor 647: Alexa-CCL2 and Alexa-CCL22; Almac Scotland, Edinburgh, UK) was added to cells. Cells were incubated for different time periods at 37 °C in 5% CO_2_ and subsequently washed twice in ice-cold FACS buffer. DRAQ7 (BioStatus, Leicestershire, UK) was added to each cell suspension to identify nonviable cells. Fluorescence intensity of the cells was acquired on a MACSquant analyzer (Miltenyi Biotec) and analyzed using MACSquant software.

##### Chemokine Degradation Assays

Cells were plated out the night before the assay into a 96-well plate to ∼80% confluency (2 × 10^4^ cells/well) in media. The following day, biotinylated human CCL2 (bio-CCL2) (Almac) was added to the media at a concentration of 50 μg/ml, and cells were incubated at 37 °C in 5% CO_2_. Medium was collected at different time points ranging from 0 to 30 h after the addition of bio-CCL2 and stored at −20 °C before being analyzed by Western blotting.

##### Immunoprecipitation of HA-ACKR2 and Sulfotyrosine Detection

HA-ACKR2 was immunoprecipitated from ACKR2-expressing HEK cells using the μMACS^TM^ epitope tag protein isolation kit (Miltenyi Biotec) with anti-HA MicroBeads. Cell lysates were prepared before magnetic labeling was performed by adding anti-HA MicroBeads to the cell lysates and incubating for 30 min on ice with regular gentle agitation. The mixture was run through a μ-column suspended on a magnet as per the manufacturer's instructions. After several washes to remove any nonspecific material, elution buffer (SDS buffer) heated to 95 °C was added to the column, and samples were collected (cell lysis buffer, wash buffer, and elution buffer are all supplied in the Miltenyi Biotec kit). Subsequent to elution samples were analyzed by SDS-PAGE before Western blotting with an anti-sulfotyrosine antibody (Millipore, Temecula, CA) or an anti-HA antibody (Abcam, Cambridge, UK).

##### Treatment with Sodium Chlorate

Cells were grown in media supplemented with a range of concentrations of sodium chlorate (40) (Sigma) for various time periods before performing chemokine uptake assays as detailed above.

##### siRNA and ACKR2 Transfection

Cells were plated out the night before in 6-well plates and grown to ∼80% confluency. The following day cells were transfected with TPST-1 siRNA, TPST-2 siRNA, or negative control siRNA using HiPerfect transfection reagent (All from Qiagen). The next day, cells were transiently transfected with the HA-ACKR2 plasmid described above using the Effectene® transfection reagent kit (Qiagen). 24 h later cells were tested for their ability to uptake AF-CCL22 using chemokine uptake assays. Cell lysates were also collected and analyzed by Western blotting using an anti-ACKR2 antibody (Sigma).

##### Quantitative PCR Analyses

Quantitative PCR was performed as described previously (17, 19) using RT kit Nanoscript (PrimerDesign, Southampton, UK) and SYBR Green mix Perfecta (QuantaBioscience, Gaithersburg, MD). The following primers were used: TPST 1 forward, 5′-GCTGGGGGAGTGTCTCTGT-3′; TPST 1 reverse, 5′-TCCGTAGTTAGGTGGGTTGG-3′; TPST 2 forward, 5′-TCGGACCTCTAATCCAAGCA-3′; TPST 2 reverse, 5′-TCCATACCCTTCATTCTCTACCC-3′; TBP forward, 5′-TGCTGTTGGTGATTGTTGGT-3′; TBP reverse, 5′-AACTGGCTTGTGTGGGAAAG-3′; ACKR2 forward, 5′-AGGAAGGATGCAGTGGTGTC-3′; and ACKR2 reverse, 5′-CGGAGCAAGACCATGAGAAG-3′.

##### Generation of ACKR2-N

Peptides representing the first 35 amino acids of the N terminus of the ACKR2 protein (ACKR2-N) were generated by peptide synthesis (Almac Scotland). Two versions of ACKR2-N were generated, with either sulfated or nonsulfated tyrosine residues. These are denoted as ACKR2-N(s) and ACKR2-N(un-s) respectively. A hexahistidine tag was incorporated into the C-terminal tail of both ACKR2-N(s) and ACKR2-N(un-s).

##### Chemokine-ACKR2-N Binding Assays

Biotinylated chemokines (Almac) were incubated with ACKR2-N(s), and the μMACS^TM^ streptavidin kit (Miltenyi Biotec) was used to ascertain any binding relationship between the two molecules. Samples collected from such experiments were then run on SDS gels and analyzed by Western blotting to examine any co-pulldown of ACKR2-N.

##### SDS-PAGE

Samples were mixed 1:4 with NuPAGE® LDS sample buffer (Invitrogen) and either heated to 95 °C for 5 min or, when analyzing ACKR2 expression, incubated at room temperature for 10 min (44). Samples were mixed 1:10 with NuPAGE® sample reducing agent (Invitrogen). 10–20 μl of each sample was loaded onto a precast NuPAGE Novex 4–12% Bis-Tris gel (Invitrogen) in a vertical electrophoresis tank (Xcell Surelock; Invitrogen) filled with NuPAGE MES SDS running buffer (Invitrogen). Novex® Sharp prestained protein standard (Invitrogen) was run alongside samples for size determination. Electrophoresis was performed for 1–2 h at 150 V.

##### Western Blotting

Gels were transferred onto a nitrocellulose membrane using an iBlot® system (Invitrogen) according to manufacturer's instructions. Following transfer, the membrane was washed in PBS Tween (PBST) and blocked in 5% Milk/PBST either for 1–2 h at room temperature or overnight at 4 °C. After blocking, the membrane was washed briefly in PBST and incubated with primary antibody overnight at 4 °C. The membrane was subsequently washed four times for 5 min each in PBST and incubated with secondary antibody for 1 h at room temperature. Blots were developed via a chemiluminescence reaction (SuperSignal WestPico kit; Pierce) before being placed between acetate sheets and exposed to x-ray film (Kodak, Carestream Health Inc., New York, NY) in a dark room for varying time periods. Film was then developed in an X-Omat processor (Konica-Minolta, Bainbury, UK).

##### Chemokine Uptake Assays with ACKR2-N as a Competitor

Cells were treated as detailed previously; however, 1 μg of ACKR2-N(s) or ACKR2-N(un-s) (Almac) was added to wells simultaneously with 60 ng/well of Alexa-CCL2 before incubation for 1 h at 37 °C in 5% CO_2_. In similar experiments Alexa-CCL2 was “precomplexed” with ACKR2-N by incubating 1.0 μg of ACKR2-N(s) or ACKR2-N(un-s) with 60 ng of Alexa-CCL2 in a total volume of 20 μl of PBS for 15 min at room temperature before addition to wells. Cells were subsequently analyzed on a MACSquant analyzer (Miltenyi Biotec).

##### Cleavage Assays with Staphopain A

Cells were plated out the night before the assay into 6-well plates to about 80% confluency (2 × 10^5^ cells/well) in regular media. The following day staphopain A (Sigma) was added to (2 μm) to wells, and the cells were incubated for various time periods ranging from 15 min to 2 h at 37 °C in 5% CO_2_. Afterward cells were scraped, and cell lysates were prepared using cell lysis buffer (Miltenyi Biotec). Samples were then analyzed by Western blotting. For chemokine uptake assays cells were washed twice with PBS before scraping off and resuspending in complete medium and adding 25 nm Alexa-CCL22. Cells were incubated for 1 h at 37 °C in 5% CO_2_ and subsequently washed twice in ice-cold FACS buffer. DRAQ7 (BioStatus) was added to each cell suspension to identify nonviable cells. The fluorescence intensity of the cells was measured on a MACSquant analyzer (Miltenyi Biotec).

##### Chemokine Fluorescence Assay Following Staphopain A Treatment

Chinese hamster ovary (CHO) K1 cells expressing ACKR2 were plated out the night before on black 96-well plates to ∼80% confluency. The following day, cells were treated with staphopain A (Sigma) in PBS at concentrations of 0.5 and 2 μm and incubated for 15 min at 37 °C in 5% CO_2_. Cells were washed twice in PBS and put back into cell growth media supplemented with 10% FCS. Alexa-CCL22 was added to the media at a concentration of 20 nm, and the cells were incubated at 37 °C in 5% CO_2_ for 1 h. Cells were washed three times with PBS and then analyzed on a PHERAstar FS fluorescence plate reader (BMG Labtech). The raw data were analyzed using the integrated MARS data analysis software (BMG Labtech).

##### Statistical Analyses

The data were analyzed using GraphPad Prism (Version 5) software (San Diego, CA) with *p* < 0.05 being regarded as significant. Individual statistical tests used are noted in the relevant figure legends.

## RESULTS

### 

#### 

##### The ACKR2 N Terminus Bears a Conserved Tyrosine Motif Involved in Ligand Binding

**FIGURE 1.**
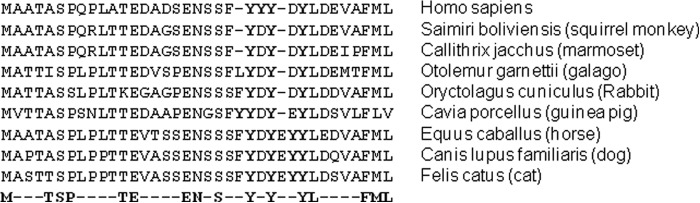
**Conservation of a tyrosine motif at the N terminus of ACKR2.** Alignment of the N-terminal sequences from the named mammalian species is shown. The tyrosine motif is highlighted in *bold type*, and the overall level of conservation at the N-terminal region of ACKR2 is shown in the *last line*.

**FIGURE 2.**
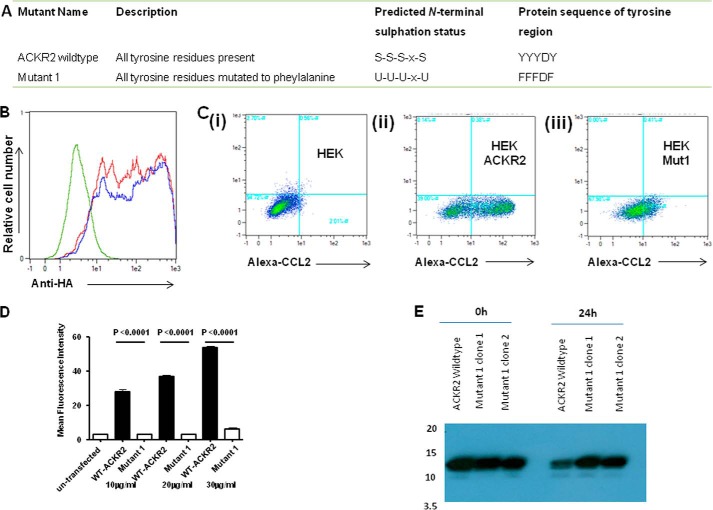
**Deletion of the tyrosine motif blocks ligand internalization by ACKR2.** *A*, in mutant 1, all the tyrosines in the identified motif (labeled *S* to indicate likely sulfation) have been mutated to phenylalanine (labeled *U* to indicate likely unsulfation). Other than this, the sequences of WT ACKR2 and mutant 1 are identical. *B*, flow cytometric assessment of the expression levels of WT ACKR2 (*blue line*) and mutant 1 (*red line*) in stable transfected HEK cells. The proteins were measured using anti-HA antibodies to detect the HA tag at the extreme N terminus. Levels of expression are reflected in the extent of the right shift in the flow cytometry profile. Isotype control antibodies demonstrate the low level of nonspecific antibody staining (*green line*). *C*, flow cytometric profiles showing the internalization of Alexa-CCL2 by untransfected HEK cells (*panel i*), HEK cells expressing WT ACKR2 (*panel ii*), and HEK cells expressing mutant 1 (*Mut1*; *panel iii*). Note the prominent right shift in *panel ii* indicative of Alexa-CCL2 binding and uptake compared with the more limited right shift apparent in *panel iii. D*, summary of mean fluorescence intensity values obtained from binding/internalization of the indicated concentrations of Alexa-CCL2 by HEK cells expressing either WT ACKR2 or mutant 1. Note that untransfected HEK cells display a very low mean fluorescence intensity value indicative of low levels of nonspecific Alexa-CCL2 binding. The data were analyzed using one-way ANOVA with Tukey's post-test. *E*, Western blot analysis of CCL2 degradation over 24 h. Biotinylated CCL2 was incubated in the presence of HEK cells expressing either WT ACKR2 or mutant 1 (results from two clones shown), and the levels of intact CCL2 remaining were assessed using Western blotting and detection by streptavidin-HRP. Note the reduction in biotinylated CCL2 levels by wild type CCR2 but not by either of the two clones of mutant 1.

Alignment of the primary sequences of the N terminus of ACKR2 from a variety of mammalian species (Fig. 1) revealed a highly conserved three-tyrosine motif that was commonly associated with neighboring acidic amino acids. Such motifs are recognized sites of conventional chemokine receptor sulfation and are important for ligand binding (32–41, 43). Because we have previously shown sulfation of ACKR2 (44), we tested the importance of this tyrosine motif for ligand binding by human ACKR2 using conventional mutagenesis. As shown in Fig. 2*A*, we generated a mutant version of ACKR2 (henceforth referred to as mutant 1) in which all three conserved N-terminal tyrosine residues, as well as a fourth unique to higher order primate ACKR2, within this motif were mutated to phenylalanines. This construct was stably transfected into HEK cells and compared with HEK cells expressing WT ACKR2 for ligand binding and internalizing ability. Both molecules had an HA tag at the extreme N terminus allowing for flow cytometric assessment of expression levels in the stable clones. As shown in Fig. 2*B*, transfected HEK cells expressed similar levels of cell surface WT ACKR2 and mutant 1. Next we assessed the relative ability of WT ACKR2 and mutant 1 to internalize ligand by analyzing uptake of Alexa-CCL2 by flow cytometry. As shown in Fig. 2*C*, it is clear that untransfected HEK cells do not internalize Alexa-CCL2 (Fig. 2C, *panel i*). However, whereas WT ACKR2 internalizes it avidly (Fig. 2*C*, *panel ii*), similar uptake is not seen with mutant 1 (Fig. 2*C*, *panel iii*). Indeed on multiple repeat experiments, shown in Fig. 2*D*, it was clear that mutant 1 displayed a significantly reduced ability to internalize ligand even at very high chemokine concentrations. Finally, we examined the ability of WT ACKR2 and mutant 1 to degrade ligand. As shown in Fig. 2*E*, whereas HEK cells expressing WT ACKR2 substantially degraded biotinylated CCL2 over 24 h, as detected by Western blotting using a streptavidin detection system, two clones of HEK cells expressing mutant 1 did not. Thus N-terminal tyrosine residues are essential contributors to the ability of ACKR2 to internalize and degrade chemokines.

##### Ligand Binding by ACKR2 Is Dependent on Receptor Sulfation

**FIGURE 3.**
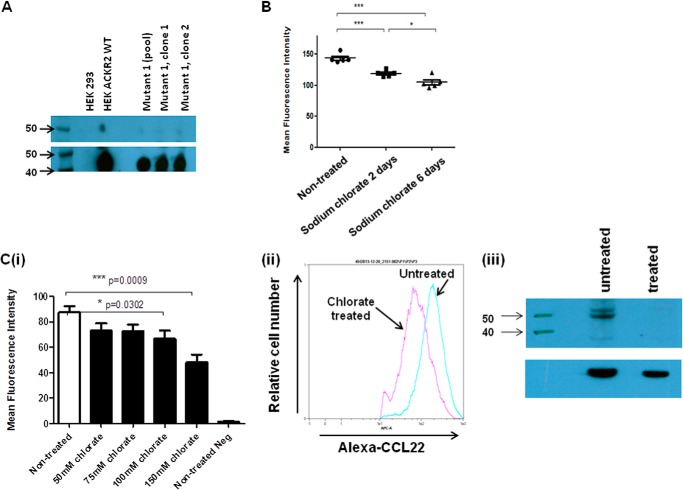
**Inhibition of protein sulfation significantly inhibits ACKR2 activity.** *A*, Western blot analysis of ACKR2 sulfation. Anti-HA beads were used to immunoprecipitate ACKR2 from a clone of HEK cells expressing WT ACKR2 and from a pool of HEK cells expressing mutant 1, as well as from two separately derived clones of mutant 1 transfectants. The 50-kDa molecular mass marker, which is coincident with migration of ACKR2 in SDS-PAGE gels, is shown in the *upper panel*. Expression was detected using an anti-sulfotyrosine antibody and loading normalized by reprobing the blot with an anti-HA antibody (*lower panel*). The 40- and 50-kDa molecular mass markers are indicated in the *lower panel. B*, mean fluorescence intensity values derived from flow cytometric assessment of Alexa-CCL22 binding to ACKR2-expressing HEK cells grown in either normal medium or medium supplemented with 30 mm sodium chlorate for the indicated times. The data were analyzed using one-way ANOVA with Tukey's post test. *C*, *panel i*, mean fluorescence intensity values measuring Alexa-CCL22 binding to ACKR2-expressing HEK cells treated with the indicated increasing concentrations of sodium chlorate. The graph also includes measurement of mean fluorescence intensity of Alexa-CCL22 binding to nontreated ACKR2 expressing HEK cells (*Non-treated*) and nontreated untransfected HEK cells (*Non-treated Neg*). *Panel ii*, flow cytometric profile demonstrating the reduction in Alexa-CCL22 binding by ACKR2-expressing HEK cells before (*blue line*) and after (*red line*) treatment for 6 h with 150 mm sodium chlorate. *Panel iii*, Western blot analysis of lysates of cells expressing ACKR2 using anti-sulfotyrosine antibodies. Cells were either left untreated or treated for 6 days with 150 mm sodium chlorate. The 40- and 50-kDa molecular mass markers are indicated. Gel loading was normalized by reprobing with antibodies to β-actin (*lower panel*).

The inability of the tyrosine mutant variant of ACKR2 to internalize ligand suggested that sulfation of these residues may, as has been reported from conventional chemokine receptors, be essential for ligand binding and uptake (32, 33, 35). We confirmed the reduction in sulfation in mutant 1 by Western blotting of immunoprecipitated ACKR2 using an anti-tyrosylsulfate antibody. As shown in Fig. 3*A*, both a pool of mutant 1 transfected HEK cells and two separately isolated clones displayed markedly reduced sulfation compared with WT ACKR2. Note that the low level staining seen in the mutant 1 tracks is seen routinely, and thus we presume it to be background. To further implicate sulfation in ligand internalization by ACKR2, we incubated HEK cells expressing WT ACKR2 in 30 mm sodium chlorate, which blocks protein sulfation, and assessed the impact of this on ligand uptake. As analyzed by flow cytometry and as shown in Fig. 3*B*, growth in the presence of sodium chlorate partially but significantly reduced the ability of HEK cells expressing ACKR2 to internalize ligand (in this case an alternative ACKR2 ligand, Alexa-CCL22 was used) in a time-dependent manner. To further examine the ability of sodium chlorate to reduce ligand binding and internalization by ACKR2, we tested higher concentrations. As shown in Fig. 3*C* (*panel i*), although concentrations of sodium chlorate as high as 100 mm partially reduced ligand binding by ACKR2, concentrations of 150 mm induced a considerably more marked and significant reduction. This was also shown by flow cytometry (Fig. 3*C*, *panel ii*) and Western blotting (Fig. 3*C*, *panel iii*). Thus both mutation of N-terminal tyrosine residues and treatment of ACKR2-expressing cells with high concentrations of sodium chlorate demonstrate the involvement of tyrosine sulfation in ligand binding and internalization by ACKR2.

##### Tyrosylsulfotransferases 1 and 2 Are Involved in ACKR2 Sulfation

**FIGURE 4.**
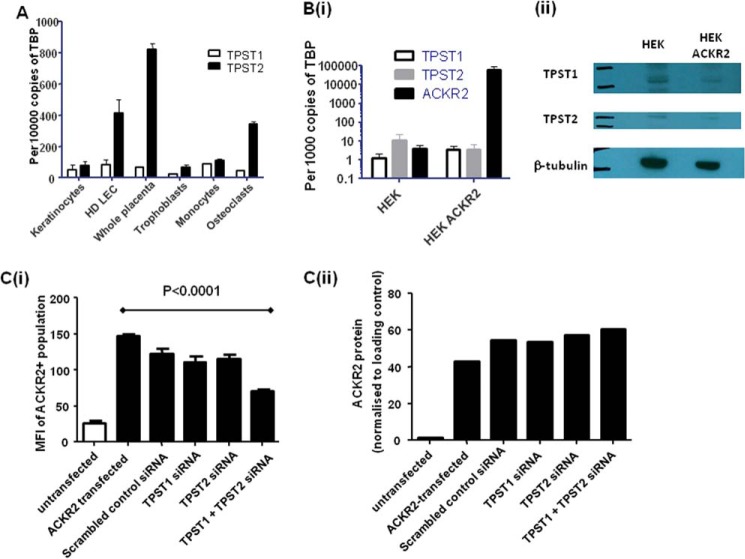
**Analysis of roles for tyrosylsulfotransferases 1 and 2 (TPST1 and 2) in ACKR2 sulfation.** *A*, RT-PCR analysis of the expression of TPST1 and TPST2 in the indicated cell types. Expression is reported as number of transcripts per 10,000 copies of TATA-binding protein (*TBP*). HD-LECs are human dermal-derived lymphatic endothelial cells. *B*, *panel i*, RT-PCR analysis of TPST1 and TPST2 expression in untransfected and ACKR2-transfected HEK cells. Expression is reported as number of transcripts per 1,000 copies of TATA-binding protein. ACKR2 transcript levels are also shown to indicate the high expression levels in transfected HEK cells. *Panel ii*, Western blot analysis of TPST1 and TPST2 protein expression in untransfected (HEK) and ACKR2 transfected (HEK ACKR2) cells. The Western blots were normalized by reprobing with antibodies to β-tubulin. *C*, *panel i*, effects of siRNA to TPST1 and TPST2 on ligand binding by ACKR2 in transfected HEK cells. The data are reported as mean fluorescence intensity values (*MFI*) of Alexa-CCL22 binding as measured using flow cytometry. Cells were treated either with siRNA to TPST1 and/or TPST2 or with a scrambled control siRNA. Mean fluorescence intensity values derived from Alexa-CCL22 treated untransfected HEK cells are included for comparison. *Panel ii*, densitometric analysis of ACKR2 expression in HEK cells treated as indicated in Fig. 4C (*panel i*) and normalized to β-actin.

Tyrosine sulfation is dependent on the expression of tyrosylsulfotransferases, of which two are known to be expressed and functioning in mammalian cells (45). To gain insights into which of these may be involved in ACKR2 sulfation, we examined their expression in tissues either associated with, or not associated with, ACKR2 expression. As shown in Fig. 4*A*, although very little expression of TPST1 was detected in any of the cell types examined, the strongest expression of TPST2 was detected in lymphatic endothelial cells and placenta, both sites of high ACKR2 expression (15–17), suggesting that TPST2 may contribute to tyrosine sulfation in ACKR2. Further analysis of TPST1 and TPST2 in HEK and HEK-ACKR2 cells revealed equivalent expression of both enzymes at the mRNA (Fig. 4*B*, *panel i*) and protein (Fig. 4*B*, *panel ii*) levels. To examine the importance of these tyrosylsulfotransferases for ACKR2 sulfation and function in transfected HEK cells, we pretreated HEK cells with siRNA to either, or both, enzymes and then transiently transfected these cells with ACKR2 and measured ligand binding (using Alexa-CCL22) 24 h later. As shown in Fig. 4*C* (*panel i*), treatment with siRNA to either enzyme individually had no significant effect on ligand binding by ACKR2; however, simultaneous treatment with siRNA to both enzymes significantly reduced ligand binding. This treatment was not associated with any reduction in ACKR2 protein expression (Fig. 4*C*, *panel ii*) and suggests that both enzymes are independently able to contribute to sulfation of and therefore ligand binding by ACKR2.

##### No Single Tyrosine Residue Is Essential for Ligand Binding by ACKR2

**FIGURE 5.**
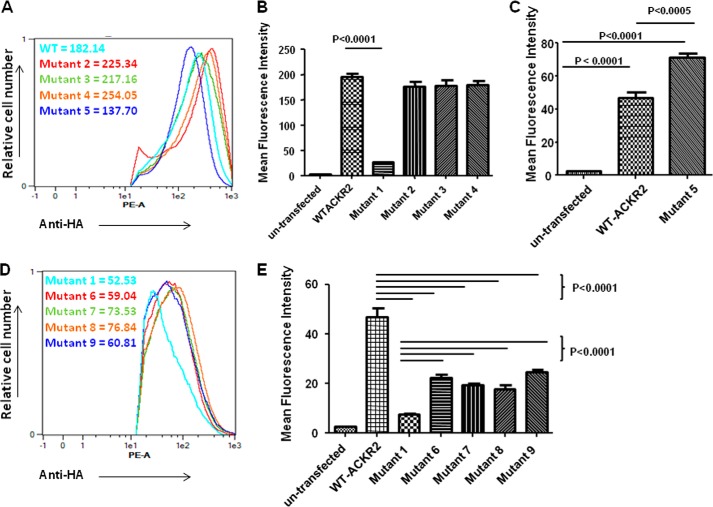
**Individual tyrosine residues do not account for the ligand internalizing activity of ACKR2.** *A*, flow cytometric assessment, using anti-HA antibodies, of the expression levels of mutants 2–5 in the transfected HEK cells. Note that the extent of the right shift of the flow cytometric profile is indicative of the extent of expression of the mutants. *B*, summary of mean fluorescence intensity values obtained from flow cytometric analysis of the binding of Alexa-CCL2 to WT ACKR2 and mutants 1–4. Note again that untransfected HEK cells do not significantly bind Alexa-CCL2. *C*, summary of mean fluorescence intensity values obtained from flow cytometric analysis of the binding of Alexa-CCL22 to mutant 5 compared with untransfected HEK cells and WT ACKR2-expressing HEK cells. *D*, flow cytometric assessment using anti-HA antibodies of the expression levels of mutants 1 and 6–9 in the transfected HEK cells. *E*, summary of mean fluorescence intensity values obtained from flow cytometric analysis of the binding of Alexa-CCL22 to WT ACKR2, mutant 1, and mutants 6–9 all expressed in HEK cells. Note again that untransfected HEK cells do not significantly bind Alexa-CCL22. All data were analyzed using one-way ANOVA with Tukey's post test.

We next performed more specific mutagenesis studies in which individual tyrosines within the conserved motif were mutated to phenylalanines (mutants detailed in Table 3). As is apparent from Fig. 1, only three of the tyrosines, tyrosines 1, 3, and 4, are conserved. Tyrosine 2 is unique to higher order primates. Each of the four tyrosines were separately mutated to phenylalanines (mutants 2–5), and the resulting HA-tagged cDNAs were stably transfected into HEK cells. As shown in Fig. 5*A*, flow cytometry for the HA tag revealed similar levels of cell surface expression of each of the four mutants. As above, ligand internalization was assessed by flow cytometry to examine the ability of the WT and mutant variants to take up Alexa-CCL2. Quantitation of multiple flow cytometry experiments (Fig. 5*B*) demonstrated again the profound inability of mutant 1, the full tyrosine variant of ACKR2, to internalize ligand. These data further demonstrated that mutation of the individual conserved tyrosine residues did not significantly impair ligand internalization compared with the WT receptor. In contrast, and as shown in Fig. 5*C*, phenylalanine mutation of the nonconserved tyrosine residue found in human ACKR2 (mutant 5) in fact led to significantly enhanced ligand internalization, suggesting that this may represent an evolutionary adaptation in human ACKR2 that alters the ability of ACKR2 to bind ligand.

**TABLE 3:** **Summary of ACKR2 mutants generated**

Name	Description	Predicted N-terminal sulfation status	Protein sequence of tyrosine region
Wild type	All tyrosines present	S-S-S-*X*-S	YYYDY
Mutant 1	All tyrosines mutated	U-U-U-*X*-U	FFFDF
Mutant 2	First tyrosine mutated	U-S-S-*X*-S	FYYDY
Mutant 3	Third tyrosine mutated	S-S-U-*X*-S	YYFDY
Mutant 4	Fourth tyrosine mutated	S-S-S-*X*-U	YYYDF
Mutant 5	Second tyrosine mutated	S-U-S-*X*-S	YFYDY
Mutant 6	Only first tyrosine present	S-U-U-*X*-U	YFFDF
Mutant 7	Only second tyrosine present	U-S-U-*X*-U	FYFDF
Mutant 8	Only third tyrosine present	U-U-S-*X*-U	FFYDF
Mutant 9	Only fourth tyrosine present	U-U-U-*X*-S	FFFDY

Next we carried out the reciprocal experiment in which we introduced individual tyrosines into the fully tyrosine mutated variant of ACKR2 (mutant 1) to see whether single tyrosine residues were sufficient to rescue ligand internalization by this mutant. These mutants were stably transfected into HEK cells as above, and as shown in Fig. 5*D*, the transfectants displayed similar expression levels as assessed by flow cytometry for the incorporated HA tag. In ligand binding and internalization studies, again using Alexa-CCL22 (Fig. 5*E*), it was clear that introduction of the single residues into the fully mutated motif is sufficient to at least partially rescue ligand uptake by ACKR2.

Thus together these data demonstrate that although tyrosine residues are essential for efficient ligand internalization, this cannot be accounted for on the basis of a single tyrosine residue within the conserved motif. Indeed, the fact that reintroduction of single tyrosines was not sufficient to fully reconstitute ligand binding and internalization indicates a requirement for multiple N-terminal tyrosine resides for full ACKR2 function.

##### A Sulfated N-terminal Peptide Derivative of ACKR2 Is Able to Bind Ligand

**FIGURE 6.**
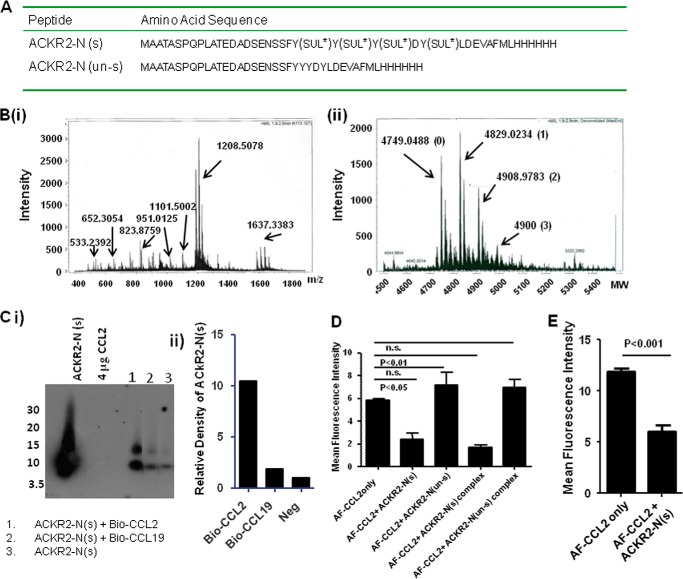
**A sulfated peptide derived from the ACKR2 N terminus binds ligand.** *A*, sequences of peptides designed from the N terminus of ACKR2 and generated by peptide synthesis. SUL* indicates a sulfatable tyrosine residue. ACKR2-N(s) represents the sequence of the fully sulfated peptide whereas ACKR2-N(un-s) indicates a fully unsulfated peptide. The hexahistidine tags are indicated at the extreme C terminus of the peptide. *B*, mass spectrometry analysis of the mixed levels of sulfation apparent in the sulfated version of ACKR2-N. The mass/charge (*m*/*z*) profile is shown in *panel i*, and the deconvoluted molecular mass profile is shown in *panel ii*. Prominent *m*/*z* peaks are indicated in *panel i*, and the molecular mass peaks corresponding to the differentially sulfated peptide variants are indicated in *panel ii*. In *panel ii*, the extent of sulfation of the different variants is indicated in *parentheses* after the molecular mass. *C*, Western blot analysis (*panel i*) using an anti-ACKR2 antibody and subsequent densitometry analysis (*panel ii*) indicate the presence of ACKR2-N(s) after pulldown binding assays using biotinylated-CCL2. As shown, the ACKR2 peptide migrates as two bands of 8 and 14 kDa. Note the markedly higher pulldown of the ACKR2 peptide by biotinylated CCL2 (*lane 1*) compared with biotinylated CCL19 (*lane 2*). The pulldown achieved by biotinylated CCL19 is not significantly different from the background binding of ACKR2-N(s) to the streptavidin beads used for the pulldown (*lane 3*). Note also the lack of nonspecific antibody cross-reacting with 4 μg of CCL2, confirming the specificity of the antibody in this assay. *D*, addition of sulfated ACKR2 peptide (ACKR2-N(s)), but not the unsulfated ACKR2 peptide (ACKR2-N(un-s)), significantly reduced uptake of Alexa-CCL2 by transfected HEK ACKR2 cells, as determined by flow cytometric analysis (data are reported as mean fluorescence intensity values from the flow cytometric analysis). The data were analyzed using one-way ANOVA with Tukey's post test. *E*, addition of ACKR2-N(s) significantly reduced uptake of Alexa-CCL2 by CCR2 expressed on THP1 cells, as determined by flow cytometric analysis. *AF-CCL2 only* refers to THP1 cells exposed to Alexa-CCL2, and *AF-CCL2*+*ACKR2-N(s)* refers to THP1 cells exposed to Alexa-CCL2 as well as the sulfated N-terminal peptide. The data were analyzed using Student's *t* test.

To further examine the importance of tyrosine residues and sulfation status for the ability of the N terminus of ACKR2 to interact with ligand, we generated a 35-amino acid peptide (ACKR2-N) corresponding to the extreme N terminus of ACKR2 (Fig. 6*A*). Two versions were generated: one was sulfated on tyrosine residues (ACKR2-N(s)), and the other was left unsulfated (ACKR2-N(un-s)). The sulfated variant was generated by tyrosine sulfation post-peptide synthesis, which yielded peptide species displaying variable tyrosine sulfation patterns (Fig. 6*B*, *panels i* and *ii*). Initial assessment of the ability of the sulfated peptide to bind ligand involved the use of biotinylated chemokine ligands and streptavidin beads to measure the ability of ligand to interact with and therefore pull down the ACKR2 peptides. This assay involves the eventual detection by Western blotting of the peptide using anti-ACKR2 antibodies. As shown in Fig. 6*C* (*panel i*) and as quantified by densitometry in Fig. 6*C* (*panel ii*), although biotinylated CCL2 was able to pull down the sulfated ACKR2-N peptide (*lane 1*), this was not seen to the same extent with biotinylated CCL19 (*lane 2*), a homeostatic CC-chemokine that does not bind to ACKR2. In fact the amount of ACKR2-N pulled down by biotinylated CCL19 was no greater than that pulled down by nonspecific interaction of ACKR2 with the streptavidin beads (*lane 3*). These data therefore confirm the relative selectivity of the peptide for inflammatory CC-chemokines.

Next we examined the ability of the peptide, in both sulfated and unsulfated forms, to interfere with the ability of the inflammatory CC-chemokine CCL2 to bind to either ACKR2 (in heterologous transfectants) or to its cognate signaling receptor CCR2 (in THP1 cells). As shown in Fig. 6*D*, the sulfated peptide, but importantly not the unsulfated peptide, was able to significantly impair binding and internalization of Alexa-CCL2 by ACKR2 transfected HEK cells. In addition, the sulfated peptide was able to significantly impair the binding of Alexa-CCL2 to CCR2 on THP1 cells (Fig. 6*E*). Notably, the ability of the peptide to inhibit ligand internalization by ACKR2 was unaffected by precomplexing with ligand (Fig. 6*D*). Importantly this precomplexing also did not enhance the neutralizing activity of the unsulfated peptide. Thus a sulfated peptide derived from the ACKR2 N terminus is capable of inhibiting ligand interactions with ACKR2 and its cognate signaling receptor, CCR2.

##### Staphopain A Can Cleave the ACKR2 N Terminus

**FIGURE 7.**
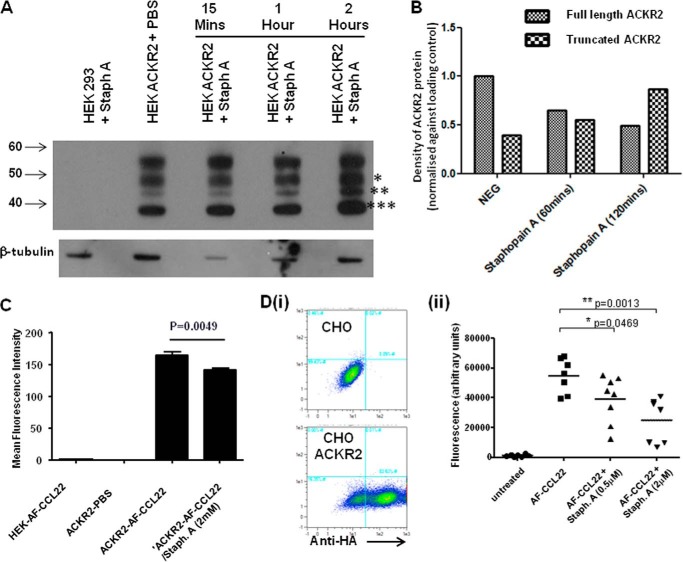
**The serine protease staphopain A cleaves ACKR2 at the N terminus.** *A*, ACKR2 expressed on HEK cells is cleaved by staphopain A (*Staph A*) as determined by Western blot analysis using an antibody specific for the C terminus of ACKR2. In this experiment β-tubulin was used as a loading control. Full-length (*) and truncated (** and ***) ACKR2 proteins are indicated. ACKR2-expressing HEK cells were treated either with PBS or with staphopain A for the indicated time points. Untransfected HEK cells treated with staphopain A are included as a negative control. The gel positions corresponding to 40, 50, and 60 kDa are indicated. *B*, densitometric analysis of band intensity from the blot shown in *A. NEG* refers to the PBS-treated ACKR2-transfected HEK cells. In this figure the full-length ACKR2 is marked with *, and the truncated ACKR2 is marked with *** in *A*. Expression levels are normalized to the β-actin loading control. *C*, flow cytometric assessment of the effects of staphopain A on Alexa-CCL22 internalization by ACKR2-expressing HEK cells. The data are presented as mean fluorescence intensity values measured from flow cytometric analysis of Alexa-CCL22 binding. *HEK-AF-CCL22* represents the levels of Alexa-CCL22 binding to untransfected HEK cells. *ACKR2-PBS* represents the background fluorescence of ACKR2 transfected HEK cells. *ACKR2-AF-CCL22* represents the binding of Alexa-CCL22 to ACKR2-transfected HEK cells. *ACKR2-AF-CCL22/Staph. A (2mm)* represents cells pretreated with 2 μm staphopain A prior to Alexa-CCL22 binding. Note, in this experiment, that the alternative ACKR2 ligand, CCL22, is used in place of CCL2. The data were analyzed using one-way ANOVA with Tukey's post test. *D*, *panel i*, flow cytometric profiles showing the expression of ACKR2 in untransfected (*upper panel*) and transfected (*lower panel*) CHO cells. ACKR2 expression levels were measured using anti-HA antibodies. The right shift of the flow cytometric profile for CHO-ACKR2 cells indicates high level expression. *Panel ii*, measurement of Alexa-CCL22 (*AF-CCL22*) binding to ACKR2-expressing CHO cells in the presence, or absence, of increasing concentrations of staphopain A. The data were obtained by measuring fluorescence intensity in a standard fluorescent plate-reader. Background fluorescence is shown by the data labeled *untreated*. The data presented in this figure are representative of more than two repeat experiments.

We have previously reported that, in heterologous transfectants, ACKR2 is subject to N-terminal processing giving rise to a 38-kDa truncated receptor that has lost approximately the equivalent of the 35-amino acid peptide generated in this study (44). There have been previous reports of pathogen-derived proteolytic enzymes cleaving the N termini of chemokine receptors, and in particular the important role of proteases such as staphopain A has been highlighted in this regard (46). We therefore exposed HEK cells expressing ACKR2 to staphopain A and observed the effects on ACKR2 over a range of time points up to 2 h. Because we were looking for N-terminal truncation, previously published anti-ACKR2 monoclonal antibodies and anti-HA tag antibodies were inappropriate because their recognition sites would have been lost. We therefore used a polyclonal antibody that detects the ACKR2 C terminus and that we have previously characterized in a range of contexts (18, 22). As shown in Fig. 7*A* and quantified in Fig. 7*B*, HEK cells expressing ACKR2 produced a variety of full-length and truncated variants detected using this antibody. Importantly one of these variants of ∼38 kDa, equivalent to the size observed previously for truncated ACKR2, appeared in increasing amounts following exposure of the cells to staphopain A. Attempts to formally confirm the molecular nature of the truncated species using mass spectrometry were unsuccessful, possibly because of the previously reported difficulties in obtaining mass spectrometry data from ACKR2 (44). In addition a number of approaches aimed at trying to purify the N-terminal peptide released after staphopain A treatment met with similar difficulties, which we presume to be a consequence of the low concentrations of peptide produced. Importantly, however, in agreement with the N-terminal truncation, a modest but significant staphopain A-dependent reduction in ACKR2 ligand (Alexa-CCL22) uptake activity was detected in staphopain A-treated HEK-ACKR2 cells using flow cytometry (Fig. 7*C*). To confirm this in a separate cell line, we transfected CHO cells with ACKR2 (Fig. 7*D*, *panel i*) and examined ligand binding and internalization following treatment with 0.5 and 2 μm staphopain A. In these experiments, ligand binding and uptake were assessed using a fluorescence-based assay adapted for 96-well plate format. As shown in Fig. 7*D* (*panel ii*), staphopain A treatment resulted in more marked inhibition of ligand uptake and internalization in this cell line than was seen with the transfected HEK cells. Specifically, at 2 μm, staphopain A was able to reduce ligand binding and uptake by ACKR2-expressing CHO cells by ∼50%. These data therefore suggest that ACKR2 is a natural substrate for staphopain A, which results in cleavage of the ACKR2 N terminus.

## DISCUSSION

Although a large number of studies have described the importance of tyrosine sulfation for chemokine receptor ligand binding and function, there have been few studies (47) into the importance of similar sulfated tyrosine motifs for atypical chemokine receptor function. This is important because atypical chemokine receptors are biochemically distinct from the signaling chemokine receptors and also typically display non-leukocyte-based expression patterns. Here we demonstrate that a conserved tyrosine motif in the N terminus of ACKR2 is essential for ligand internalization and scavenging and that within this motif, multiple tyrosines are required for efficient ligand uptake. Thus the importance of this motif is maintained in the atypical chemokine receptor subfamily. Formal demonstration of the importance of sulfation of these tyrosine residues is presented in the form of sodium chlorate inhibition. Growth of HEK cells expressing ACKR2 in the presence of chlorate significantly reduced ligand internalization by ACKR2. However, this was incomplete at concentrations of sodium chlorate typically used in such experiments (30 mm), and this probably relates to the extremely long half-life of the ACKR2 protein (13, 48). Notably, use of higher concentrations of sodium chlorate (150 mm) resulted in more substantial and highly significant inhibition of ligand uptake and internalization by ACKR2-expressing HEK cells. Importantly the roles played by sulfated tyrosines are further confirmed by the use of peptides derived from the ACKR2 N terminus. Notably a sulfated variant of the peptide is able to bind ligand and thereby inhibit ligand binding to ACKR2 or the signaling receptor CCR2, whereas the unsulfated variant is not. The ability of a peptide from the ACKR2 N terminus to bind ligand suggests that this site is of general importance for chemokine ligand binding to receptors because similar peptides from other conventional chemokine receptors also display ligand binding properties (33, 38, 49, 50). Overall, these data indicate that the sulfated tyrosine motif is essential for ACKR2 function and that a peptide derived from this area is able to bind ACKR2 ligands.

We have previously reported that, in cells expressing ACKR2, the protein is detected in two processed forms. The first is the full-length protein, and the second is an N-terminal variant truncated by ∼35 amino acids. We have proposed that this may represent shedding, from the seven-transmembrane base of ACKR2, of a peptide capable of binding ACKR2 ligands and such a function of this peptide is supported by the current study. This peptide may therefore act in the vicinity of ACKR2-expressing cells as a motile blocker of inflammatory CC-chemokine function. Thus far we have not identified any mammalian proteases capable of generating such a peptide from intact ACKR2. However, previous reports of the ability of bacterially derived proteases to cleave the N terminus of other chemokine receptors, most notably CXCR2 (46), prompted us to examine whether such proteases can also cleave the ACKR2 N terminus. Here we provide evidence of truncation of ACKR2 by the *Staphylococcus aureus* protease staphopain A to yield an N-terminally truncated variant of ∼38 kDa. In keeping with the importance of this region for ligand binding, staphopain A treatment of ACKR2-expressing cells also significantly reduced ligand internalization. This reduction was modest in ACKR2-expressing HEK cells but was considerably more marked in ACKR2-expressing CHO cells. These differences may relate to the relative recycling ability of ACKR2 from the extensive intracellular stores in HEK cells and CHO cells but may also be explained by differences in cell surface glycocalyx density and thus access for the enzyme to ACKR2. Despite this functional evidence of truncation, which supports the Western blot data, attempts at obtaining mass spectrometry confirmation of the molecular nature of the truncated ACKR2 product were unsuccessful. We have previously reported difficulties in mass spectrophotometric analysis of ACKR2 (44) and propose that this is likely to be the reason for the current difficulties and may relate to the specific nature of the truncated product resulting from staphopain A treatment. In addition we used a number of approaches to try to identify the released N-terminal peptide including immunoprecipitation with anti-HA antibody. Again this proved unsuccessful, suggesting either that the concentration of released peptide was very low or that the peptide was further degraded in the culture medium. Assuming the ability of staphopain A to release an intact N-terminal peptide from ACKR2, the question then is why might a bacterially derived protease wish to cleave the N terminus of ACKR2? Cleaving the N terminus of CXCR2 makes sense because this will neutralize neutrophil migration over which, with the exception perhaps of some murine contexts (51), ACKR2 has little influence. The possible reason for staphopain A cleavage of ACKR2 is that this will, in fact, complement the CXCR2 cleavage by yielding small peptide inhibitors of inflammatory CC-chemokine function, thereby preventing recruitment of myelomonocytic inflammatory leukocytes. Importantly, although the overall impairment of ACKR2 function associated with staphopain A cleavage was modest, this may be of little concern to the pathogen if generation of small amounts of N-terminally derived peptide is all that is required.

The ability of the ACKR2-derived peptide to inhibit the binding of CCL2 to its cognate receptor, CCR2, suggests that this may have some therapeutic benefit. This would be particularly important if the ACKR2-derived peptide was able to bind all ACKR2 ligands. Notably, despite it being over two decades since the initial cloning of chemokine receptors, there are no small molecule blockers of chemokine receptor function licensed for use in inflammatory pathologies. This inability to therapeutically utilize inflammatory chemokine receptor blockers most likely reflects the highly redundant nature of the inflammatory chemokine response (52, 53). In terms of inflammatory CC-chemokines, many are produced simultaneously at inflamed sites. This is why the promiscuity of ACKR2 and its ability to internalize and scavenge all inflammatory CC chemokines make it such an efficient neutralizer of inflammatory CC-chemokine function. The advantage of the N-terminal peptide over individual small molecule receptor-blockers would lie precisely in its ability to neutralize the activity of multiple different inflammatory CC-chemokines. This is the strategy adopted by a number of viral species (42), and the proper therapeutic advancement of such a strategy may be of major importance in the development of novel anti-inflammatory therapeutics. However, the ACKR2-derived peptide in its current form does not yet display sufficient affinity for ligand to merit *in vivo* testing.

In conclusion, therefore, we provide for the first time evidence of the importance of N-terminal tyrosine residues for ACKR2 function. We highlight this area as being essential for ligand internalization and scavenging and demonstrate the ability of a peptide derived from this region to bind inflammatory CC-chemokines.

## Footnotes

2 The abbreviations used are:

ACKR: atypical chemokine receptors
ANOVA: analysis of variance.
